# Toxic effects of per- and polyfluoroalkyl substances on sperm: Epidemiological and experimental evidence

**DOI:** 10.3389/fendo.2023.1114463

**Published:** 2023-02-20

**Authors:** Zhangbei Sun, Yiqian Wen, Binhui Wang, Shiyi Deng, Fan Zhang, Zhendong Fu, Yangyang Yuan, Dalei Zhang

**Affiliations:** ^1^ School of Basic Medical Sciences, Nanchang University, Nanchang, China; ^2^ Clinical Medical Experimental Center, Nanchang University, Nanchang, China; ^3^ Jiangxi Provincial Key Laboratory of Reproductive Physiology and Pathology, Nanchang University, Nanchang, China

**Keywords:** per- and polyfluoroalkyl substances, reproductive toxicity, sperm, testosterone, male fecundity

## Abstract

As emerging organic contaminants, per- and polyfluoroalkyl substances (PFASs) have aroused worldwide concern due to their environmental persistence, ubiquitous presence, bioaccumulation, and potential toxicity. It has been demonstrated that PFASs can accumulate in human body and cause multiple adverse health outcomes. Notably, PFASs have been detected in the semen of human, posing a potential hazard to male fecundity. This article reviews the evidence about the toxic effects of exposure to PFASs on male reproduction, focusing on the sperm quality. Epidemiological studies showed that PFASs, such as perfluorooctanoic acid (PFOA) and perfluorooctane sulfonic acid (PFOS), were adversely associated with the semen parameters in humans, including sperm count, morphology and motility. Experimental results also confirmed that PFAS exposure led to testicular and epididymal damage, therefore impairing spermatogenesis and sperm quality. The mechanisms of reproductive toxicity of PFASs may be involved in blood-testosterone barrier destruction, testicular apoptosis, testosterone synthesis disorder, and membrane lipid composition alteration, oxidative stress and Ca^2+^ influx in sperm. In conclusion, this review highlighted the potential threat of exposure to PFASs to human spermatozoa.

## Introduction

The decline in human fertility rates has drawn considerable concern (1, 2). Accumulating evidence suggests that human semen quality has decreased worldwide over the past few decades (3–9). Although the causative factors remain to be fully discovered, exposure to environmental pollutants is considered to be a major contributor for impaired male fecundity (2, 10–12). Per- and polyfluoroalkyl substances (PFASs) are a family of fluorinated synthetic chemicals that have been extensively used in industry and consumer products since the 1950s. As emerging persistent organic contaminants, PFASs are extremely resistant to environmental degradation and metabolic clearance due to their unique and stable physicochemical properties (13). Consequently, these compounds are ubiquitous and persistent in the environment and accumulate in the food chain (14, 15), posing a serious threat to ecological and human health worldwide. A variety of PFASs have been detected in the semen of human (16–19), implying a potential hazard of PFASs to male fecundity. Hence, this review briefly summarizes the epidemiological and experimental evidence regarding the toxic effects of PFAS exposure on male reproduction, focusing on sperm quality.

## Human exposure to PFASs

Accumulating evidence has revealed that humans are universally exposed to PFASs (20–24). The intake of polluted food and drinking water, inhalation of indoor air and dust, and dermal contact are claimed as the major routes of human exposure to PFASs (14, 25–27). Perfluorooctanoic acid (PFOA) and perfluorooctane sulfonic acid (PFOS) are the most predominant and frequently detected PFASs in human blood (28). Their serum half-lives were estimated to be 3.8 and 5.4 years in human body, respectively (29). In human plasma, PFASs primarily bind to albumin and are transferred through the body (30). Epidemiological investigations have indicated a possible association between PFAS exposure and adverse health outcomes, such as liver function abnormality (31), glucose homeostasis disturbance (32), dyslipidemia (33), cardiovascular diseases (34), fetal growth restriction (35), and bone mineral density reduction (36).

## Effect of PFASs on human sperm quality and quantity

### Sperm concentration and count

In a previous investigate on 105 Danish males from the general population, sperm concentration and total sperm count showed a reduced tendency in those with high PFAS levels, although not at statistically significant levels (37). Similarly, a nonsignificant decrease was observed in 212 young men from the PFASs-polluted Veneto region (38). However, a recent investigation on 864 young males from the general Danish population showed that maternal exposure to PFASs was linked to lower sperm concentration and total sperm count (39). A multivariable linear regression analysis on 169 male offspring also demonstrated that *in utero* exposure to PFOA was related to lower sperm concentration and total sperm count (40).

### Morphology

Sperm morphology is an important determinant of semen quality and male fertility. Epidemiological studies have shown that exposure to high levels of PFASs are associated with a lower percentage of morphologically normal spermatozoa (37, 38). An investigation on the partners of pregnant women from arctic and European populations showed a 35% decrease in the proportion of sperm with normal morphology in those with the highest PFOS exposure relative to those with the lowest exposure (41). Furthermore, a Longitudinal Investigation of Fertility and the Environment (LIFE) study reported that perfluorooctane sulfonamide (PFOSA) was related to smaller sperm head area and perimeter and a higher percentage of bicephalic and immature sperm, and PFOA, PFOS, perfluorodecanoate (PFDeA) and perfluorononanoate (PFNA) were associated with a lower percentage of sperm with coiled tails (42).

### Motility

Sperm motility is a decisive factor for male fecundity. In the population of the Pearl River Delta region in China, a significantly negative correlation was observed between sperm motility and PFASs in semen (17). Consistent with this finding, in a cross-sectional study, seminal PFOS, PFOA and emerging chlorinated polyfluorinated ether sulfonate (6:2 Cl-PFESA) were associated with a decline in the percentage of progressive sperm and an elevation in the percentage of DNA fragmentation (18). In addition, maternal exposure to PFASs was also linked to a higher percentage of immotile and nonprogressive sperm in the young adulthood (39). In our previous study, *in vitro* exposure to PFOA conspicuously impaired the capability of human sperm to penetrate artificial cervical mucus (43). Similarly, *in vitro* incubation with PFOA led to a remarkable reduction in progressive motility in human sperm (44).

### Capacitation, acrosome reaction and hyperactivation

Sperm capacitation, acrosome reaction and hyperactivation are essential prerequisites for the fertilization of oocyte. However, epidemiological data regarding the effects of PFASs on these processes are scarce. Our previous study showed that incubation with PFOA *in vitro* compromised progesterone-induced acrosome reaction and viscous medium penetration in human sperm (43). Similarly, *in vitro* exposure to PFOA and PFOS decreased the number of capacitated spermatozoa and hindered progesterone-induced acrosomal reaction in boar spermatozoa (45, 46).

### Experimental evidence for toxicities of PFASs to sperm

Numerous studies have confirmed the male reproductive toxicities of PFASs in rodents. It has been shown that PFOA exposure causes epididymis injury and reduces epididymal sperm count in mice (47–49). Furthermore, sperm motility and progressiveness were remarkably compromised and teratospermia rate was significantly elevated in PFOA-treated mice (48). Correspondingly, rats exposed to PFOS also displayed a prominent decline in epididymal sperm count and sperm viability and motility, concomitant with a notable increase in the percentage of morphological abnormalities of head, mid-piece and tail of sperm (50). In another study, PFOS exposure at 5 mg/kg did not affect the number of sperm or the percentage of motile sperm, but significantly reduced the motility of sperm reflected by decreased curvilinear, straight-line and average path velocity in mice (51). In addition, exposure to PFOA and PFOS induced male reproductive toxicity in *Caenorhabditis elegans*, leading to a reduction in spermatid size and motility and an increase in sperm malformation rate (52). Chronic exposure to PFOS also decreased sperm density and compromised the total and progressive motility in zebrafish (53).

## Discussion

Due to the environmental persistence and pervasive presence, there is growing concern regarding the toxicities of PFASs to male fertility. Numerous studies have suggested that PFASs induce testicular toxicity. For example, exposure to PFOA repressed the expression of blood-testis barrier (BTB) proteins and increased TNFα content and p-p38/p38 MAPK ratio in mouse testis and cultured Sertoli cells (54). Similarly, hexafluoropropylene oxides and PFOS disturbed BTB by activating p38 MAPK/MMP9 pathway (55, 56). These results indicate that p38 MAPK signaling may contribute to PFASs-induced BTB disruption. Proteomic profile analysis also indicated that PFOA treatment altered blood-testis barrier remodeling in mouse testis (57). Furthermore, PFOS exposure promoted the generation of reactive oxygen species (ROS) and suppressed the activities of antioxidases in the testes, thereby impairing testicular physiology and spermatogenesis in rats (50). Oral PFOA administration resulted in the destruction of the seminiferous epithelium, induced oxidative stress, inhibited NRF2-mediated antioxidant response, and led to apoptosis in the testis of mice (49, 58). However, studies found that supplement with flavonoids rutin and pachypodol attenuated testicular damage caused by PFOA and PFOS through alleviating oxidative stress, respectively (49, 50). Additionally, maternal exposure to PFOA reduced serum testosterone levels, disrupted testis development, damaged testicular structure, and caused testicular apoptosis in the offspring mice (59, 60). These results suggest that exposure to PFASs can result in the disruption of testicular structure and function, which may be partly responsible for PFASs-caused reduction of sperm count. Moreover, antioxidative intervention with flavonoids may be a promising strategy for preventing and rescuing PFASs-induced spermatogenic impairment.

Hormones in the hypothalamic-pituitary-gonadal axis are important regulators in the reproductive process. Intratesticular testosterone plays an important role in sperm number and sperm motility, and abnormal testosterone generation impairs spermatogenesis in humans (61). PFASs have been identified to act as endocrine disruptors affecting male reproductive health. A cross-sectional study reported that higher serum levels of PFASs are negatively associated with testosterone concentrations among male adolescents (62). The negative association between serum PFOS and testosterone levels was also observed in healthy young Danish men (63). In laboratorial experiments, PFOS exposure disrupted the hypothalamic-pituitary-testis axis activity (64, 65), and reduced luteinizing hormone, follicle-stimulating hormone and testosterone levels in adult male rats (50). Furthermore, both PFOA and PFOS significantly decreased the expression of steroidogenic enzymes and the concentrations of testosterone in male mice (48, 66), and the mechanisms may be involved in developmental inhibition, oxidative stress and apoptosis in Leydig cells (67–70). Inversely, low-dose PFOA stimulated steroid hormone synthesis by enhancing fatty acid metabolism and steroidogenic activation in Leydig cells (71). These results suggested that exposure to PFASs disordered testosterone biosynthesis, which may be correlated with the impaired semen quality.

Normal sperm function is crucial for male fertility. Some epidemiological and experimental investigations also showed a negative association between PFAS exposure and semen parameters, such as sperm count, morphology and motility, implying that PFASs have an adverse influence on sperm quality (**Tables 1** and **2**). Nevertheless, the toxicological mechanisms remain largely unelucidated. Rodent studies demonstrated that PFOA could accumulate in the epididymis and cause morphological change in epididymal epithelium (47, 49), suggesting that the epididymis is a potential target and PFOA may exert direct toxicity to spermatozoa. Lu et al. (47) found that PFOA exposure activated AKT/AMPK signaling pathway, altered polyunsaturated fatty acid composition, and triggered oxidative stress in the epididymis of mice (47). Furthermore, *in vitro* treatment with PFOA augmented ROS production and reduced sperm viability (47). The study implied that oxidative stress and membrane polyunsaturated fatty acid alteration may be involved in PFOA-induced sperm toxicity. Moreover, PFOA incubation resulted in accumulation in sperm membrane, and perturbed plasma membrane fluidity, mitochondrial respiratory activity and electrochemical potential, indicating that PFOA impacts human sperm motility by plasma membrane disruption (44). Our previous study showed that *in vitro* PFOA exposure compromised the penetration ability of human spermatozoa by inducing oxidative stress, evoking CatSper-mediated Ca^2+^ influx, and compromising progesterone-induced response (43). Testicular transcriptome profiling revealed that PFOS exposure led to alterations in microtubule-based movement, microtubule motor activity, cilium movement, cytoskeleton and spermatid development (51). In addition, PFOS altered sperm membrane lipid composition reflected by elevated ratio of cholesterol to phospholipids in male zebrafish (53), suggesting that PFOS may sperm function through disrupting membrane fluidity. These findings may help explain the abnormalities in sperm morphology and motility caused by PFASs.

**Table 1:** Epidemiological studies on toxicities of PFASs to sperm.

PFASs Type	Samples	PFASs content (ng/mL)	Note on impact	Reference
PFHxS PFOA PFOS	Blood	6.6 (4.0-12.1) 4.9 (2.7-7.2) 24.5 (14.2-42.1)	Lowered sperm concentration and total sperm count.	(37)
PFHxS, PFHpA, PFOA, PFOS, PFNA, PFDA, PFUnDA	Semen	0.77, 0.06, 4.4, 27.56, 0.38, 0.15 and 0.12, respectively.	Combined maternal exposure reduced sperm concentration and total sperm count, and increased the proportions of nonprogressive and immotile sperm.	(39)
PFOA	Blood	3.8 (2.8-4.7)	*In utero* exposure lowered sperm concentration and total sperm count with higher FSH and LH levels.	(40)
PFOS, PFOA, PFHxS, PFNA	Semen	18.4, 3.8, 1.1 and 1.2, respectively.	Increased sperm morphology defects.	(41)
Σ9PFASs	Blood Semen	160 17	Decreased sperm motility.	(17)
PFOA, PFOS, 6:2 Cl-PFESA	Serum	8.57, 8.38 and 6.09, respectively.	Increased the percentage of progressive sperm and the percentage of DNA fragmentation.	(18)
PFOA, PFOS, 6:2 Cl-PFESA	Semen	0.23, 0.1 and 0.06, respectively.		
Me-PFOSA-AcOH, PFDeA, PFNA, PFOA, PFOS	Semen	Michigan: 0.4, 0.3, 1, 4.6 and 19.15, respectively; Texas: 0.25, 0.5, 1.65, 5.3 and 21.6, respectively.	PFOSA reduced sperm head area and perimeter and increased the percentage of bicephalic and immature sperm. PFDeA, PFNA, PFOA and PFOS reduced the percentage of sperm with coiled tails.	(42)

**Table 2:** Experimental studies on toxicities of PFASs to sperm.

Species	PFASs	Doses	Note on impact	Reference
Human	PFOA	0.25, 2.5 and 25 mg/mL	Compromised P4-initiated sperm migration and acrosome reaction, reduced sperm penetration ability, and induced sperm oxidative stress.	(43)
Human	PFOA	0.1-10 ng/mL	Increased the percentage of non-motile sperm, altered membrane fluidity, and reduced sperm motility.	(44)
Boars	PFOA/PFOS	500, 1000, 2000 and 3000 µM	Increased sperm mortality, and affected sperm capacitation.	(45, 46)
Mice	PFOA	1.25, 5 and 20 mg/kg/d	Reduced epididymis weight, altered polyunsaturated fatty acid composition, and induced oxidative stress.	(47)
Mice	PFOA	0, 0.31, 1.25, 5, and 20 mg/kg/d	Reduced sperm quality, damaged seminiferous tubules, and reduced testosterone and progesterone levels.	(48)
Mice	PFOA	20 mg/kg/d	Disrupted epididymal epithelium.	(49)
Rats	PFOS	20 mg/kg	Increased sperm mortality, and reduced sperm viability and epididymal sperm.	(50)
Mice	PFOS	1 and 5 μg/g	Decreased epididymal sperm motility.	(51)
*Caenorhabditis elegans*	PFOA/PFOS	0.001, 0.01 and 0.1 mmol/L	Reduced spermatid size and motility, and increased the rate of malformed spermatids.	(52)
Zebrafish	PFOS	0, 5, 50, and 250 μg/L	Reduced sperm density and the total and progressive motility, and changed sperm membrane lipid composition.	(53)

## Conclusions

We have summarized the toxicological effects of PFASs on sperm using recent epidemiological and experimental data. Increasing evidence suggests that exposure to PFASs is adversely associated with sperm quality, and their mechanisms of toxicity might be involved in testicular and epididymal damage, testosterone synthesis disorder, and oxidative stress, membrane lipid composition alteration and Ca^2+^ influx in sperm. However, due to the limited number of epidemiological studies, the reproductive health risk of human exposure to PFASs, especially their emerging alternatives, needs further investigation.

## Author contributions

ZS and YW: Writing-original draft preparation, writing-review and editing. BW and SD: Writing-review & editing. FZ and ZF: Data collection, conceptualization. YY: Writing-review & editing, conceptualization. DZ: Supervision, writing-review and editing, funding acquisition. All authors contributed to the article and approved the submitted version.
